# A Practical Treatise on Urethritis and Syphilis

**Published:** 1836-04-01

**Authors:** 


### Medico-Chirurgical Review,

### No. XLVIII.

[No. 8 of a Decennial Series.]

JANUARY 1, to APRIL 1, 1836.

### A Practical Treatise on Urethritis and Syphilis: including Observations on the Power of the Menstruous Fluid, and of the Discharge from Leucorrhœa and Sores, to produce Urethritis: with a Variety of Examples, Experiments, Remedies, and Cures. A new Nosological Classification of the various Venereal Eruptions; each Order illustrated by Coloured Plates, and exemplified by numerous Cases; and a Proposal of a Substitute for Mercury. By *William Henry Judd*, Surgeon in His Majesty's Fusileer Guards, &c. &c. Octavo, pp. 568.

We cannot compliment Mr. Judd upon his title-page. It is more like an index, or a table of contents, than what a title-page should be. Did we not know Mr. Judd to be a highly respectable surgeon, we should suppose we were perusing the announcement of some popular work by Mr. Goss or Mr. Van Butchell—gentlemen so humane as to be ever on the watch to inform the public where the most unmentionable maladies may be safely, surely, and expeditiously cured, "without caustic, cutting, or confinement." Mr. Judd is evidently unused to authorship, and we do not doubt that he will avoid this unintentional error on a future occasion.

Those who are best acquainted with the venereal disease, will probably be least surprised at the appearance of a new work upon the subject. There is no disease in which the practical and experienced surgeon observes a more decided and irreconcileable discrepancy between what he has read, or has been taught, and what he actually sees. Sores exist in books, as separate and individual things, for which he looks in vain in Nature—and she, on the other hand, displays phenomena which he never finds in books. The besetting sin of most writers on syphilis has been theory. The very last work which has been published on the subject has been ruined by the spirit of speculation that pervades it. The author has almost invariably been employed in fitting the disease to his Procrustian bed.

It would occupy us too long to enter on the causes which have rendered the various works on the venereal either useless or injurious. It is certain that, for the most part, they convey a very erroneous idea to the inexperienced reader of the nature, the characters, and the treatment of the malady. Some are less objectionable, and some more so, but a good treatise on syphilis and gonorrhœa there is not. It is to supply this acknowledged defect that Mr. Judd has produced the volume before us. From the opening of his preface, it will be obvious that our author comes forward as a nosologist.

"No work (he says) having hitherto issued from the press, so arranging and classifying the various symptoms and numerous eruptions produced by *Urethritis* and *Syphilis,* that the physiological student, or medical attendant, might at once refer to it in order to ascertain the Order, Genus, and species to which any particular venereal affection of the skin belongs;—to learn what has been the general effect of the many curative modes of treatment hitherto employed in each stage;—to enable himself to premise, *à priori,* the probable duration of the affection, and to ascertain what usually have been the suffering and disorganization caused by it, and the termination of the disease in such cases; and the want of such assistance, as also of a nomenclature on the same subject having been frequently experienced by myself during my early studies; I was induced to collect (during the last twenty years) the various notes and cases that form the principal basis of the following pages." viii.

The work is divided into two parts. The first is subdivided into two portions;—the first on urethritis venerea, or gonorrhœa—the second on the cuticula externa, or, in other words, an anatomical description of the skin. The second part is broken into five chapters. Of these, the first is on the origin and nature of the venereal disease—the second, on the variety of primitive sores and absorbent surfaces—the third is made up of observations on the various forms of syphilitic eruptions—the fourth is on the most inveterate secondary symptoms—and the fifth is on mercury, and "*on a new remedy*" where mercury fails.

A large, indeed by far the largest, portion of the work consists of cases, usually reported with diurnal details, and arranged without any very apparent regularity or method. In the section, for example, on urethritis, the history and account of the disorder occupy four pages, and the reports of cases ninety-five—a proportion closely resembling the ratio of the sack to the bread in Falstaff's bill.

We shall now proceed, without further preface, to the work itself. Where we differ from the author, we shall freely express our dissent, because we believe that too many of the notions propagated and prevailing on the subject of venereal maladies are erroneous, or actually pernicious.

We observed that four pages only are devoted to the subject of gonorrhœa, or, as Mr. Judd prefers to term it, urethritis venerea. We regret this, because there is no good account of this venereal affection. Many of its concomitants, and some of its consequences, have not been noticed, or, if noticed, they have been so very imperfectly. Yet, short as Mr. Judd's description is, we differ widely from him in many particulars.

Urethritis, he says, is a primary syphilitic affection, and is tantamount, except as to the structure it invades, to a venereal sore, and even becomes one when it causes, which it occasionally does, ulceration of the lining membrane within the urethra.

This is the revival of the old notion of the identity of syphilis and gonorrhœa. Mr. Judd does not attempt to tell us why there should be such very broad distinctions between the phenomena, the progress, and the consequences of the two diseases: unless the assertion, indeed the assumption, that it is because the syphilitic virus is in the case of gonorrhœa applied to the mucous membrane, be considered as the explanation in question.

Let us look at this hypothesis a little more in detail. Syphilis affecting the mucous membrane of the urethra is said by these gentlemen to be strangely modified by the nature of the tissue. This might be plausible, if the mucous membrane exerted the same influence in other situations. But undoubtedly it does not. If the syphilitic virus be applied to the lip, we never heard of its occasioning a *labiitis*, but it gives rise to a very orthodox chancre. If the matter of gonorrhœa comes in contact with the mucous membrane of the eye, it occasions in it an inflammatory purulent discharge—the gonorrhœal ophthalmia. But in cases of secondary symptoms, secondary syphilitic ulcerations occur in the conjunctiva, and we have twice seen syphilitic primary sores at its margin.

The mucous membrane of the vagina is the seat of both diseases—gonorrhœa and syphilis. Those who pin their faith on the paramount influence of tissue would seem to have forgotten this altogether. Yet gonorrhœa and syphilis are as distinctly marked in women as in men. When we look at all these circumstances we must admit how idle the supposition is, that it is the tissue which makes syphilis in the mucous membrane assume the characters which we know by the name of gonorrhœa.

It will be observed that Mr. Judd makes urethritis become a venereal sore, when it causes ulceration of the mucous membrane. But this is obviously a petitio principii, for he has to prove that it is urethritis which does produce ulceration of the mucous membrane. On this point we join issue with him, for we believe, that it is not simple gonorrhœa, but real syphilis. The argument may be thus stated:—as, in ninety-nine cases out of the hundred, gonorrhœa evinces no tendency whatever to occasion ulceration of the mucous membrane, and as in the hundredth case such ulceration may happen, sometimes with and sometimes without the ordinary gonorrhœal symptoms, it is easier to suppose that this is a superadded affection, than that gonorrhœa should so change its ordinary characters.

Mr. Judd proceeds to state very explicitly in what he conceives urethritis to consist.

"Urethritis is an inflammatory affection caused by the irritation of the syphilitic virus from a pustule on the delicate mucous membrane lining the urinary canal, or vagina. Generally its specific action produces a discharge of pus only: but, at other times, it also produces a pustule, or abscess, within the urethra, similar to what it more usually does on the surface, and just like the discharge from a pustule on the glans penis; for the pustule is caused by the same secretion as the urethritis, having its origin with it in common from a venereal pustule, sore, or discharge, in the vagina; and it is the product of pus from either of these ailments." 2.

When we read the statement that urethritis was primarily a pustule on the mucous membrane, we were naturally curious to see the evidence on which that statement is made. But we must own that we cannot find any,—not even the shadow of a proof. We have never seen such pustules. When patients present themselves with gonorrhœa we cannot discern them, and in two examinations of the urethra which we have had an opportunity of making after the individual's death, we have found nothing save augmented vascularity of the mucous membrane, for two or three inches down the urethra. We must therefore regard the preceding position as at present quite unsupported.

Mr. Judd states that the syphilitic virus sometimes produces a pustule or abscess within the urethra, similar to what it more usually does on the surface. Unfortunately, the language of our author is ambiguous, and we feel at a loss to understand his precise meaning. But we were not aware that the syphilitic virus occasioned an abscess on the surface. In cases of secondary symptoms abscesses occur, but at this moment we do not recollect a familiar instance of abscess occasioned by a primary sore. The following is the case adduced, of abscess produced by the syphilitic virus in the urethra.

*Case.* William W——te. August 26th, 1818.—Urethritis, which commenced three days after contamination, and also a large swelling situated just under the integument of the body of the penis, and about one-third of its length from the glans.

Pil. Extr. Col. C. c̄. Calomelane.

27th.—The swelling filled with pus, made its way through the corpora cavernosa, and broke internally into the urethra, where it gave out four ounces of blood. Since the abscess burst, the swelling has much diminished, and there is less tenderness on pressure; but pain is felt as the urine flows. This man got well in September without the abscess opening externally.

Now this was clearly a common example of abscess occurring in the cellular tissue of the penis. In inflammatory gonorrhœa, the inflammation constantly extends to the contiguous tissues. Every now and then it spreads to the cellular tissue, and occasions suppuration. But this is an accidental, not a necessary consequence of the gonorrhœa, a result of the concomitant inflammation, not a part of the specific disease. In cases of common inflammation of the tonsils, we not unfrequently have suppuration in the cellular tissue of the palate, the inflammation spreading from the tonsil to it. Abscess of the penis is a case exactly analogous. Mr. Judd has committed the common error, of not separating the inflammatory attendants on gonorrhœa, from the disease itself. The former are simple inflammatory complications—the latter a specific disorder.

The last passage in this debateable paragraph, contains the unqualified affirmation, that syphilis and gonorrhœa have the same common origin from a venereal pustule, sore, or discharge in the urethra. The proof which our author offers is a case which we shall quote.

*Case.* "Mr. W. May 20th, 1825.—Three days after a suspicious connexion, a small round pustule, about the size of a pin's head, with much surrounding hardness, was observed upon the penis, just below the glans.

Three or four days afterwards, a second pustule formed near the first, and they were very similar both in size and appearance.

Mr. W. now learned that this woman's first paramour was laid up with venereal sores.

Whilst the two ulcers were healing, Mr. W. was visited by another female, an old acquaintance, and, in the course of her visit at his lodgings, he had not sufficient command to resist her importunities, and actually removed the lint, and had connexion with this second woman. The last-named female had no ailings (that she ever knew of) produced by the above impure connexion, and therefore she somē days afterwards had connexion with another gentleman, an acquaintance of mine.

June 15th.—The latter gentleman has applied for advice (in a fright), having pains in both groins, and some enlarged glands in one of them. As he had no sore, it was very doubtful what source of irritation had caused his inguinal glands to enlarge. Leeches and cold lotion were applied, and he was purged.

18th.—The enlarged glands had no sooner been put back than he was attacked by urethritis; hence it is probable that irritation in the urethra had caused the previous pains and enlargement of glands.

There are several inexplicable circumstances attending these occurrences, which led me to keep an account of them. In the first place, no secondary symptoms followed Mr. W——'s sores; and this unfortunately leaves us uncertain as to their nature being strictly venereal, although he obtained them from a woman whose associate was at the very moment laid up with what the medical attendant considered to be venereal sores, and for them he put him under mercurialization. In the next place, how did the second female, so improperly had connexion with, escape, when he had the two open sores upon him? Lastly, is it possible that the discharge from Mr. W——'s sores was conveyed through the second woman without affecting her; and yet, that the virus from them produced enlarged glands and urethritis in the third person? The facts lead to the following query:—Can a discharge from venereal sores, when applied to the urethra of another person, produce a discharge resembling, or actually being urethritis? I have ample reason to believe that it can, and could offer many cases in proof. And, be it observed, a sore on the verge of the urethra produced discharge, resembling gonorrhœa, in the case herein related of John Brown, he being at the time confined to bed, and certainly he had had no connexion for many preceding weeks; and this is by no means a solitary case." 24.

It will be observed, that the very important position which our author lays down, is supported by only indirect and objectionable evidence. A female's "paramour" has venereal sores. The female herself has connexion with a gentleman, who gets pustules, not followed by secondary symptoms. He has connexion with another female, who is not infected; and she has connexion with another gentleman, who contracts gonorrhœa. Now there is no evidence to prove that either female was diseased; the only conclusive evidence to establish the fact of the pustules in the first gentleman being really syphilitic sores—that is, the occcurence of secondary symptoms, he not being treated with mercury, is wanting also; it is a mere supposition to argue that the matter of venereal sores produced both syphilis and gonorrhœa, when the syphilis is dubious, and the source of the gonorrhœa absolutely uncertain; and finally, the whole chain of evidence is too brittle and too broken to be received as any thing more than a curious train of circumstances, the elucidation of which would require more strict investigation than was made, or, probably, would have been permitted. Testimony in venereal cases is fallacious in the highest degree. The assertions of gentlemen may usually be depended on, because they have nothing to gain by deceit, and truth is with them both a sentiment of honour and a habit. But the testimony of men in the lower classes, and, we regret to say, of females in all, is deserving of far less attention. This is especially the case with the description of females that we have to deal with in venereal cases. In them we usually look in vain for any thing like sincerity or candour. Their habits, inclinations, often their subsistence are grafted on successful falsehood. They constantly deceive without an apparent object; and where their *character* is at all involved, we must reckon on nothing but thick and thin mendacity. Such is the portrait that experience must draw of the class of females who retail syphilis and gonorrhœa. It would be unsafe to take the hour of the day upon their evidence. It is absurd to support doctrines by their testimony. In the case we have been considering, what proof can be offered, that the second female had connexion with no one, between the *known* intercourse with the first gentleman on the one hand, and with the second on the other? No proof, save the word of a whore.

On the whole, we cannot say that Mr. Judd has done any thing to establish this frequently advanced, and frequently rejected supposition—the identity of the poisons of syphilis and gonorrhœa. Time and accurate experiments may eventually prove it. At present, evidence and probabilities oppose it. The phenomena are different, the consequences are different, and the treatment is different.

Mr. Judd proceeds to state, that—

"It is very common for the same person to be attacked by urethritis venerea, and by a pustule, or syphilitic sore, about the time the discharge appears from the urethra, and both caused by one and the same connexion.

The external disease often commences by a pustule, and so situated in the male, that at times it may have been seen to break, to ulcerate, and to reach the lips of the urethra, and thereby its specific discharge to produce puriform secretion from the mucous membrane; in fact, constituting urethritis." 3.

The case to which Mr. Judd refers is the following:—

"John B——n. July 9th, 1832.—A few days after connexion, he found a deep, clean sore between the glans and prepuce. His bowels were cleared by purgatives, and he then underwent a mercurial course. His mouth was kept tender from the 14th of July to the 13th of August. The sore gradually healed at its posterior edge, and extended at its anterior one, until it reached the lips of the urethra, and there it is causing some inflammation.

15th.—A thin discharge has commenced from the urethra, accompanied by scalding, and sensations exactly similar to that of gonorrhœa.

Capiat Bals. Copaibæ ℳ xvj. ter die.

Lotio Plumbi Acet. dilut.

18th.—He has less scalding, but the running continues, and it has become thicker. The sore was dressed within the lips of the urethra with dry lint.

25th.—The orifice of the urethra is swollen and red, and both the discharge and scalding are increased, so as exactly to resemble the symptoms of common urethritis.

Low diet, quiet in bed.
Lotio Plumbi Acet. dilut.

30th.—The irritation has lessened, and both the sore and the urethra are less inflamed.

Solut. Cupri Sulph. comp.

Bals. Copaibæ ℳ xx. ter die.

October 4th.—The sore has healed, but there still is some discharge from the urethra.

8th.—The urethritis has ceased, and he is quite well." 187.

In the preceding case a sore spread from the surface of the glans into the urethra, and occasioned scalding, and discharge from within it. That this was urethritis, that is, inflammation of the mucous membrane, there can be no question. But it does not therefore follow, that it was specific gonorrhœa. Discharge from the urethra and scalding in the act of micturition may be produced by many causes independently of the agency of a specific poison. An excess in diet will occasion it—we have seen it succeed an attack of lithiasis—a blow will cause it—so will the introduction of foreign substances into the urethra, as a bougie ; the retention of a catheter in the bladder, almost invariably inducing it—excessive venery may give rise to it, independently of any disease in the female. One of the most obstinate cases of urethritis we ever had to treat, was after the removal of the nucleus of a lithic acid calculus from the urethra. We employed a pair of forceps to crush the stone, and necessarily irritated the urethra a good deal. Now if discharge and pain in micturition be considered, per se, as conclusive proof of gonorrhœa, then gonorrhœa may certainly be occasioned by whatever irritates the urethral mucous membrane. But how will this agree with the doctrine, that the poisons of gonorrhœa and of syphilis are identical ? This is one of the dilemmas into which the supporters of that doctrine tumble. We should say that, in the case of Mr. Judd, the ulcer at the orifice of the urethra gave rise to urethritis, as any other cause of irritation and of inflammation might have done. To deny this it would be incumbent on him to prove that it was a genuine gonorrhœa, that is, that it occasioned that disease in another individual.

It is by no means uncommon for a syphilitic ulcer to invade the orifice of the urethra. If we have seen that once, we have seen it, at the least, a dozen times. But we have no hesitation in asserting, that it usually does *not* give rise to urethritis. On this point of fact we can speak positively—at least we can do so with respect to the cases that have fallen under our own observation. The most frequent result that we have witnessed, has been a certain degree of contraction at the orifice of urethra. We could relate, were it necessary, half a dozen cases, in which decided syphilitic ulceration at the orifice of the urethra was unattended with urethritis.

If a syphilitic sore, extending to the urethral mucous membrane, occasions urethritis, why does not a syphilitic sore do the same thing in the vagina. If only men existed, we might understand the doctrine of the difference of tissue occasioning some of the distinctions between gonorrhœa and syphilis. But, in women, the same tissue is the seat of both, yet he must be a bungling surgeon, or a very inexperienced one, who cannot recognize the differences between gonorrhœa and syphilis in them.

"When the virus," continues Mr. Judd, "produces urethritis, and pustules or abscesses, in the membranous lining of the urethra, the breach of surface at times leads to secondary venereal symptoms, as venereal pustules or sores on the surface more commonly do." 3.

We understood that urethritis was always produced by "the virus." Why, then, do not secondary symptoms usually follow ? When we look over Mr. Judd's cases, we see him treat this syphilitic urethritis by copaiba, not by mercury. How is it, then, we repeat, that secondary symptoms are so rare? Mr. Judd seems to imply, that these occur when pustules or abscesses are formed in the urethra. We have looked over Mr. Judd's cases, and we find none to inform us what pustules in the urethra are—nor where they are—nor how they are discoverable. In the absence of this indispensable information, we must pause before we can admit an assertion, unsupported by even the offer of proof.

We have already shewn that abscess in the urethra is a misnomer. It is abscess of the cellular tissue external to the urethra. As this is merely the result of inflammation, it is not, *à priori*, likely that secondary symptoms should follow it. But we have something better than à priori reasoning to lead us to deny that secondary symptoms do follow it. We have seen many cases of abscess of the penis as a consequence of gonorrhœa—the patients were not treated by mercury, and in *no one instance* did secondary symptoms ensue.

"I am aware," concludes Mr. Judd, "that some still believe the matter of syphilis, and that of gonorrhœa (as they call it), to be distinct poisons: but I cannot overlook the well-known fact, that a woman, having a pustule or sore in the vagina, can cause urethritis in one man, and a venereal pustule or sore in a second, that also has connexion with her; and this within a few minutes of each other; although, upon examination, she is found to have a pustule or sore only, or simply an elytritis, and perhaps no perceptible discharge at the moment from either. When, again, a pustule on the penis, and an attack of urethritis in the male, are common results from one connexion,—and with a female, who on examination is seen to have elytritis only;—when a man who has connexion with her, on the third or fifth day, suffers an attack of urethritis, and has within the week a pustule or sore within his urethra, and in some instances, after a lapse of about six weeks, followed by pains, sore throat, eruptions, and confirmed secondary symptoms, bearing exact similitude to the product of the true venereal pustule: these peculiar coincidences are, I take it, proof enough and quite sufficient for the identity of the poisons.

I know that by a few I shall be censured, and accused of leading my readers to disbelieve what by some have been considered orthodox facts. To them I would reply by presenting for their consideration such cases as those of G—— W——l, William G——e, and Charles P——n; and there drawing their attention to the suppurating sores formed by abscesses or pustules within the urethra; to the enlarged glands; to the nocturnal pains, inflamed synovial membranes, lichen, ecthyma, and psoriasis, that every now and then follow the infection through the urethra." 4.

Let us look at the cases advanced by Mr. Judd.

The first is that of a man, C. B——n, who, after connexion with a prostitute, had a sore on the penis, near the pubes, and urethritis also. Mercury was not used, and secondary symptoms followed. According to the man's account, both sore and discharge proceeded from the same connexion, four days previous to their appearance. This case proves of course, supposing the statement of the man to be correct, that one prostitute gave rise both to syphilis and gonorrhœa. But how feeble an argument in favour of the identity of the poisons this is, we think we shall speedily convince our readers. The common class of prostitutes comparatively seldom have syphilis alone. At the Lock Hospital, we almost always find that both diseases coexist. The fact is, that few women have been long upon the town without having chronic discharge. These women disregard gonorrhœa. After the acute symptoms are over, they generally let it run on; and when they contract syphilis, it is, in the majority of cases, superadded to gonorrhœa, acute or chronic, which was in the vagina already. After the syphilitic sores are cured, the discharge usually remains, for it seldom or never yields to the anti-syphilitic treatment. In this state women leave the hospital—indeed they cannot usually be got to remain. Knowing, as we do, these circumstances, our wonder is, not that syphilis and gonorrhœa occasionally coexist in men, but that they co-exist so seldom. The argument founded on that coexistence is in itself worth nothing.

2. Mr. Judd argues on a suppositious case, at least we must presume it to be suppositious, for we do not see it. The case we allude to is this—that a woman with gonorrhœa only gives a man urethritis, followed, in a week, "by a pustule or sore within his urethra," and, "in some instances," by pain, sore throat, and eruptions. When the case is brought before us, we may take it into consideration—at present it only exists in the form of a petitio facti, and Mr. Judd cannot in reason require us to entertain it seriously.

3. Mr. Judd presents to the consideration of sceptics "such cases as those of G—— W——l, William G——e, and Charles P——n." Let us see what these cases really prove. We will take them in the order in which they are arrayed.

*Case* 1. G. W——l, Jan. 1826. He had urethritis, which lasted an unusual length of time, in spite of all remedies. The discharge at last ceased, but was followed immediately by pains resembling rheumatism both in his knees and legs, and so severe, as to produce lameness.

This was the third urethritis he had had, and in all three instances followed by similar pains; and in each attack they continued during several months after the discharge had ceased.

This case, so far as we can read its characters, is surely one of gonorrhœal rheumatism, and of nothing more. That such rheumatic symptoms do occasionally follow gonorrhœa is not, so far as we know, denied. But gonorrhœal rheumatism differs, in many essential particulars, from the secondary symptoms of syphilis, and its avowed peculiarities are rather evidence against than for the identity of the diseases.

*Case* 2. "William G——e, May 22d. Three months since he had urethritis, which continued a fortnight. He is now thickly studded over with lichen upon his fore-arms, thighs, and legs: some are just desquamating. A few of them appear to contain lymph, but have little inflammation round their base. He states positively that he has 'not had a sore these five years,' nor is there any mark as if he had.

Hyd. Submur. c̄. Pulv. Jalapæ.

Ung. Hyd. ʒj. omni nocte illinendâ.

June 23d.—His mouth is sore; he has used twenty-four drachms of mercurial ointment, and the eruption is quickly disappearing. He went into the country for change of air and recovery of strength.

July.—He returned well at the end of three weeks." 50.

*Case* 3. P— C——s, January 14th, 1819. Four months since he had urethritis, and he positively states without either sore or excoriation. There is now lichen—inflammation of the conjunctiva—and a sore throat. As the lichen disappeared, the skin and scalp desquamated, and the greater part of the hair fell off. He was put on blue-pill and sarsaparilla. In February, he had a second eruption, somewhat resembling psoriasis—the throat was still tender—and there were fissures about the verge of the anus in a red and half-secreting state. At the end of February, he is reported to have been well.

*Case* 4. Charles P——n, April 2d, 1827. In the previous week he had urethritis. This suddenly ceased, and the testicle became inflamed. On the 26th it was better, but on the 28th it re-inflamed. On the 13th of May the prepuce became swollen. On the 19th, he had some discharge from the urethra, and hardness within on its lower part, about half an inch from the orifice. On the 2d of June, an abscess broke into the urethra. On the 4th, a crop of lichen came out, the throat was inflamed, a gland in the neck was enlarged, and there were symptoms of pyrexia. On the 28th, there was another attack of pain in the limbs, and some return of the inflammation of the testicle. On the 3d of July, a fresh eruption of lichen. On the 6th of September an impetigo appeared on both legs. He was now ordered to take the Plummer's pill twice daily, and decoction of sarsaparilla. On the 9th, fresh lichen and papulæ appeared, and "the submaxillary glands on the right side were enlarged."* On the 11th, iodine was substituted for the Plummer's pill. On the 18th, this was changed for bark and soda. On the 1st of October, the abscesses in the neck were opened. On the 18th, a fresh crop of lichen appeared. In November, another gland swelled in the neck. The bark was discontinued, and liquor potassæ was given. On the 14th of January he was reported well.

In another case, urethritis was followed in three months by pains in the limbs, an eruption of "ecthyma," and loss of flesh. "Each large pustule on the legs ulcerated, and the cellular membrane formed sloughs and tedious ulcers that cicatrized slowly."

Such are the cases of secondary symptoms from gonorrhœa related by our author—such is the evidence to which he appeals, and appeals with confidence, as establishing the identity of that complaint and syphilis. On these cases, and on the arguments founded on the occurrence of gonorrhœal secondary symptoms, we would offer the following observations.

1. It will be observed that, in two of the preceding cases, the patient *stated* that he had laboured under gonorrhœa only, and that he had had no sore nor excoriation. Such evidence is exceedingly fallacious and unsatisfactory. Superficial sores at the angle behind the corona glandis are constantly overlooked by patients. We have repeatedly seen them present themselves with discharge from within the prepuce, and suppose that they were labouring under gonorrhœa when either the latter had no existence, or was combined with sores or excoriations in the spot that we have mentioned.† Nay, we have seen patients apply on account of true secondary symptoms, and deny that they had ever any primary symptoms at all. We have at this time a gentleman under our care, with tubercular eruption and

* Mr. Judd must here allude to the lymphatic glands. The submaxillary salivary gland, which is frequently said to be enlarged, and to suppurate, is very seldom indeed affected. But it is contiguous to several lymphatic glands, which are frequently inflamed.

† We have now under our care a respectable tradesman, who came to us on account of his hair falling off, ulceration of the throat, and syphilitic psoriasis. He said that he had gonorrhœa three months previously, but denied that he had had a sore. We asked him to draw back the prepuce, and on its inner fold discovered a well-marked indurated cicatrix. On drawing his attention to it, he observed, that he then remembered his having "a pimple" there, but he thought it was of no consequence, paid no attention to it, and, until we pointed out the scar, had quite forgotten the circumstance.

yellow ulceration of the throat, who positively states that he is not aware of having had either gonorrhœa or sores, and attributes his complaint to a blow on the testicle and violent inflammation of that organ received in hunting. This gentleman can have no object in deception, and either we must say that he is himself deceived, or that syphilitic secondary symptoms have occurred independently of syphilis—which is the more probable conjecture we need hardly say.

The surgeon must recollect that a syphilitic sore may exist in the urethra. The following cases, of which we shall merely mention the heads, have come under our own observation.

*Case* 1. An out-patient of the Lock Hospital presented himself with what we considered common gonorrhœa. We employed what we thought the appropriate remedies, but still the discharge remained. At the end of six weeks or two months, an attack of pyrexia ushered in yellow ulceration of the throat, and syphilitic psoriasis. We thought that we had met with an unequivocal instance of secondary symptoms after gonorrhœa. But, on carefully examining the urethra, we found on the inferior surface of the penis, about an inch or rather less from the orifice, a circumscribed induration, of about the size of a silver two-penny piece, evidently seated in the corpus spongiosum urethræ, and conveying to the feeling precisely the impression which an indurated sore on the surface presents. On making pressure the patient complained of pain, and pus tinged with blood escaped from the orifice of the urethra. We should observe, that the characters of this induration were very different from those of enlargement or suppuration of a lacuna. In the latter case, the hardness resembles what a pea might produce. It is small, distinct, and globular. But in this instance, the hardness had a larger area and was flat. We immediately concluded that we had met with a syphilitic sore in the urethra, and put the patient on a course of mercury. The induration rapidly subsided, the discharge pari passu diminished and ceased, and the secondary symptoms were gradually removed.

*Case* 2. About six months ago, a gentleman consulted us for what he considered a clap. He had been for four or five months under the care of a distinguished surgeon in London, and had run the gauntlet of the orthodox remedies for gonorrhœa. Cubebs, copaiba, the turpentines, and injections of all sorts had been tried, and, we believe, bougies had been employed.

On examination we found that a profuse thin discharge escaped from the urethra, with much scalding and pain in the act of micturition. On everting the lips of the orifice as much as possible, we could plainly distinguish, a little within it, yellowish ulceration of the mucous membrane. The urethra was felt distinctly indurated for an inch or more from the orifice, when pressure was made from without; and that pressure occasioned discharge and was very painful.

Now in chronic inflammation of the corpus spongiosum, there is induration of that structure, and pain on pressure. But in chronic inflammation of the corpus spongiosum, there is no ulceration of the mucous membrane. We should observe, too, that in that affection the induration is more uniform than it was in the case we are detailing. These circumstances induced us to suspect at once that we had to do with syphilitic ulceration in the urethra. In prosecuting our inquiries we ascertained that this gentleman had ulceration, of a yellowish colour, of the tonsils, and enlargement of them; and, independently of common pityriasis, with which his back and breast were covered, he had patches of true psoriasis mingled with it.

We determined to put our patient on a course of mercury. Under this he rapidly improved. The ulceration of the throat healed, the psoriasis passed away, the pityriasis, however, remaining—the induration of the corpus spongiosum disappeared—the ulceration of the mucous membrane healed—and then the discharge ceased altogether. In fact, this gentleman was completely cured by a mercurial course, and is now under our care for a common gonorrhœa, which he has recently contracted.

3. It is not enough that "secondary symptoms" follow gonorrhœa. To prove the identity of this and syphililis, those secondary symptoms ought to be identical. Perhaps the most decided and unequivocal gonorrhœal secondary symptom, is what has been denominated gonorrhœal rheumatism. We see no such peculiar affection as a consequence of syphilis. In cachectic cases we not unfrequently observe inflammation of the synovial membranes. But these cases differ widely from those of inflammation of the same membranes in "gonorrhœal rheumatism."

The purulent ophthalmia, which we sometimes observe as a concomitant or a consequence of gonorrhœa, independently of any ascertained direct application of the gonorrhœal matter, is not observed to follow syphilis.

A wide distinction must be drawn between many of what are too commonly described under the general term of secondary syphilitic eruptions. The pustular eruptions are frequently cachectic. We have twice seen an eruption of ecthyma, that could not be distinguished from the secondary syphilitic eruption, occur in dropsy, when the patient had neither laboured under syphilis nor gonorrhœa. Impetigo very frequently occurs on the lower extremities of patients of the meaner class, while affected with venereal symptoms. It is removed however by rest, purging, and such tonics as sarsaparilla, and it would not be proper to set it down as a secondary eruption. In one of the cases detailed by Mr. Judd, ecthyma and ulcerated legs are reported as the consequences of gonorrhœa. The considerations we have mentioned would lead us to pause before we attached much importance to those symptoms.

4. But taking all the preceding arguments and facts into the account, we have no doubt that secondary symptoms which cannot be distinguished from those of syphilis, occasionally follow gonorrhœa. Yet observe the difference which even here exists between syphilis and it. In syphilis the occurrence of secondary symptoms, if mercury be not employed, is the rule—in gonorrhœa it is not only the exception, but the rare exception. We would appeal to the common sense of the profession on this point. How many cases of clap must every surgeon witness—how few instances of secondary symptoms from it he sees! This is the broad view of the question which very seldom deceives.

5. It might be supposed that those gentlemen who argue so strongly for the identity of syphilis and gonorrhœa, and for the frequency of secondary symptoms in the latter, would be consistent in their views of treatment, and employ mercury for both complaints. But this is not the case with Mr.

Judd. He makes no observations on the treatment of gonorrhœa, but, from his cases, we collect that he uses copaiba, injections, and means of that description, much in the ordinary way. Now this is a practical admission of a distinction between the two disorders, and, whatever may be said or whatever may be done, we venture to pronounce that Nature will not change, however doctrines may, and that whatever theory be up or down, clap, as a general rule, will not require a course of mercury to prevent secondary symptoms, whilst syphilis certainly will.

We observed before, that Mr. Judd offers no observations on the principles that should regulate our treatment of gonorrhœa, and, therefore, we pass on to the second part of the volume, which treats of the venereal virus.

Our author's observations are too desultory to enable us to attempt to analyse them. We must content ourselves with alluding to the following subjects.

1. Mr. Judd refers to the institutions of Moses, as proof of the antiquity of the venereal disease. We must say that we look upon this dispute as exceedingly unprofitable. It is impossible now to settle the question; and, if it were possible, it would be useless. It matters little to the present generation, whether the Jews, in the days of the Pharoahs, were poxed or not; and if we understand what syphilis is, and the best way of curing it, we may pocket our ignorance of whence it came, and how it was originally generated.

2. A more important point to be determined is, whether syphilis can originate, independently of the agency of ready-formed syphilitic matter. Mr. Judd is a warm advocate of this spontaneous or independent generation of the malady. His arguments seem to be chiefly these.

"Let us fancy," he says, "an insulated colony, peopled by clean, healthy, and undefiled persons, and let us consider whether, in their social intercourse, such a disease could arise, and without any contagion being brought amongst them by new comers. It will, I take it, be readily allowed that all animal bodies are at times prone to take on some morbific action or other; and it may be observed that but few persons are exempt from a pimple every now and then, or some little breaking out (as it is termed) on the face, breasts, shoulders, etc. and, believe me, the penis and vagina are liable to a similar eruption, in common with other parts:* indeed, a vesicle or pustule, or an occasional abrasion, will often form on the corona glandis, labia, etc., or even ulceration take place, from friction, or from acrid secretions, especially if they are permitted to remain and become putrid, within the foreskin or labium. Now, we will suppose a man gets a tear during sexual intercourse; well, if his constitution is not in possession of health, is it not likely enough that a sore with unhealthy discharge will take place, and form such an ulcerated surface? And this is more apt to happen from the acrid nature of the pus formed about the sebaceous lacunæ. Then again, the female vagina, on the very next night, may become the depository of this discharge; and should this woman have intercourse with a variety of other men, one of them may come to her with a pimple on his glans, and on the succeeding

---

* "May 1st, 1835. I examined about six hundred persons, and in three instances found vesicles on the parts of generation, in individuals that also had vesicles at the time on their faces: and some of these were among married men."

day another with a vesicle or pustule—each act of connexion probably not only breaking this minute vesicle or pustule, but also squeezing out its contents into the vagina. Well, when these retained discharges become rancid, they excoriate the parts of the woman in whom they have lodged, and a breach of surface, or probably an ulcer, will then be formed from the irritation of the lining membrane; and a sore, or a discharge, will of course be produced in the next person who may chance to have intercourse with her. Besides, it is well known that secretions from the vagina (and of a kind, we have no right, in the common view of the subject, to consider venereal) often produce sore-throat, ulcers in the palate, and eruptions. (Vide case of Charles P——n, etc.)" 140.

It will be observed that this is throughout a suppositious case, and cannot logically or reasonably be admitted as any proof that what is supposed is true. Mr. Judd refers to the instance of C. P——n, as confirmatory of the view that secretions from the vagina, of a kind we have no right to consider venereal, may produce sore throat, ulcers in the palate, and eruptions. On referring to that case, we find it simply stated that the patient had urethritis, not a word being said of the origin of the disorder. We must, therefore, presume that it was contracted in the ordinary way.

Now if simple vesicles, pustules, and so forth, produce thus readily the venereal disease, it is singular that, in some way or other, it happens that few married men or women are infected, unless they forsake the connubial bed, and do what they should not. Amongst the thousands of married people in whom the fair proportion of vesicles, pustules, and so forth, must occur, we very seldom find that syphilis springs up, unless there is a good reason for it. A man when he marries never counts on syphilis as one of the inconveniences of matrimony, but he *does* count on syphilis as one of the inconveniences of promiscuous intercourse. Nothing is more common than herpetic vesicles on the penis, and on the outskirts of the female organs; yet it has not been our fortune to see these vesicles in honest people turn to pox. We say that this is a circumstance that should make us look on the spontaneous origin of syphilis with doubt. That spontaneous origin will usually be found to be extra-connubial; and, if it has nothing to do with infection from specific morbid matter, it is a better instance than we usually meet of direct and positive retribution.

One thing is quite certain—that inflammation and suppuration of the mucous membrane of the urethra and vagina, may arise from many causes, independently of specific contagion. The complaint thus produced is not distinguishable from true gonorrhœa. Whether it is or is not infectious, has not yet been settled by sufficient and satisfactory experiments; but there are reasons for believing that it is.

Gonorrhœa appears capable of giving rise, in certain individuals, and especially in women, to condylomata. Condylomata again occasion, if they exist for a length of time, peculiar secondary symptoms. Condylomata which ulcerate upon the surface, appear to be capable of generating similar sores in a cutaneous surface, exposed to the influence of their secretion. For these facts we can vouch, and, so far as they go, they appear to establish the spontaneous origin of sores which are communicable, and occasion secondary symptoms. Yet we need not observe how vague this evidence is, nor need we state, what all must feel, that our present information with respect to the independent production of syphilis, must be looked on as rather negative than positive.

3. Mr. Judd believes that syphilis is not a simple, but a compound disease—in short that there is a plurality of poisons, as there is undoubtedly a plurality of sores. This is a very reasonable opinion, and one which is most consistent with the facts. We see sores of different characters, which run a different course, require varieties of treatment, and evince differences in the mode and the degree in which they contaminate the system. It is almost impossible to suppose that all can result from one poison. But Mr. Judd goes farther than this, and contends that the very same sore results from a mixture of several poisons, and is the medium by which those several poisons are conveyed into the system. He grounds this idea on the occurrence of more than one form of secondary eruption in the same individual.

"If," he remarks, "the venereal were a simple and not a compound virus, i. e. consisting of many poisons, why should it produce a greater number or variety of eruptions from one source than any other virus that we are hitherto acquainted with? The pure lymph from cow-pock produces the vaccine vesicle only; and pure pus from small pox the variola vesicle only; and their compound or mixed virus produces modified small pox only, and so with all other poisons from every known source. If the venereal consisted of a single virus, it should invariably arise from a similar sore, and always produce one effect, as lichen only, or rupia only; or ecthyma only, and not papular eruption followed by vesicular, and those by pustular, and all arising out of the same virus. If I believed in this opinion, that all these varied symptoms were the produce of one virus, I might with equal propriety expect to produce peas and beans on the same peduncle,* and from one seed pea. Yet I do not mean to deny that a single poison is not at times secreted, or that a single vitus is not at times imbibed; and the latter circumstance probably does occasionally take place even when several are presented to the absorbents, for they are known to possess an elective attraction, (or at least they take up alkalies and refuse acids,) which is well illustrated by several of the cases herein related, that occurred in my own practice. Doubtless two persons may be infected by various secondary symptoms by the same woman, even on the same evening: for the one may imbibe a different poison to the other: indeed there are considerable grounds for believing that the absorbents of certain individuals will always take up one peculiar kind of virus in preference to another; and thus some have one peculiar form of ulcer or eruption produced, however often they may contract venereal disease, and however many poisons they may be exposed to. Hence, you may observe the same patient applying for advice with a similar set of symptoms during a series of years, although produced by many fresh contaminations, and from association with fresh individuals from distant districts."† 153.

Thus, if we admit the soundness of Mr. Judd's opinions, we may suppose that one woman may give one man one description of sore, another man another sore, and a third a different one, and finally, that she may give to a fourth, or a fifth, or a sixth lover, a sore which shall be composite and contain several or all the morbid poisons. The infection depends, in this view of the case, on the elective powers of the absorbents in the indi-

---

* A term used in Botany for a stem.

† We cannot help observing what appears to be inconsistency on the part of Mr. Judd. He argues very earnestly for the plurality of syphilitic poisons, because there are some differences in the phenomena, while he denies the distinction between gonorrhœa and syphilis, in which those differences are much more remarkable.

viduals affected. When we couple this doctrine with the previous one, that the various sores may be readily originated from common vesicles, discharges, and so forth, we must confess that our author supplies us with a very efficient machinery for the propagation and perpetuation of pox.

Although we believe in the plurality of venereal sores and of venereal poisons, we cannot go so far as our author has gone. We do not deny that it may be as he says, but we have not yet seen satisfactory proof that it is so.

It is difficult to imagine that one sore should be a Pandora's box, containing within it many poisons. The consideration which would seem to weigh most with Mr. Judd, is the occurrence of more than one form of secondary eruption in the same individual. He cites the cases of vaccinia and small pox, in which only one form of secondary eruption follows the inoculation of the morbid poison. We must pause before we receive this as a conclusive argument.

Analogical reasoning should be employed with judgment and received with caution. No two facts are exactly alike, and the deduction obtained from one, must only be used to illustrate, not establish, the deduction from another. Analogy is peculiarly fallacious when applied to the explanation of diseases which arise from a specific poison. Their very nature isolates them in a great degree, and withdraws them from the control of general, to place them under that of their peculiar laws. The laws of the origin, development, and progress of small-pox, measles, scarlet fever, plague, all differ. One morbid poison is communicable by inoculation, as the small-pox—another, measles, is not. Temperature exerts sueh an influence on some, as on plague, that it only exists within a certain range—whilst other morbid poisons, as small-pox, scarlet fever, syphilis, prevail in all climates, under all temperatures, and in every season. Plague and syphilis occasion bubo ; measles and scarlet fever do not. It would be idle to push these observations farther. Each specific morbid poison displays phenomena, and obeys laws of its own; each disease produced by it must become a special study. Even if it were true that no other morbid poison occasions more than one description of secondary eruption, that would not prove tha one syphilitic poison cannot do so. That fact is to be determined or refuted not by ascertaining what small-pox does, but what syphilis itself does. Let us see how the case stands.

A man, after connexion, has a sore which exhibits certain characters. We will say, for instance, that it displays the well known features of the Hunterian chancre. This sore is followed by a tubercular eruption and a yellow ulceration of the throat. Another man has a sore to all appearance similar to the preceding. In him that sore is succeeded by lichen, and that lichen is followed, perhaps, by rupia. Now in these two cases, which are not imaginary, we find what appears to be the same description of primary sore occasion not only different secondary eruptions in different individuals, but different eruptions in the same individual. The question then comes to be simply this :—Shall we trust the evidence of sense—or shall we disregard the obvious fact, and because small pox is thought to occasion one eruption only, deny that one syphilitic poison can occasion more ? This is the dilemma. For our own parts, we prefer to read the book of nature in the plainest sense, and in the present condition of our knowledge, to believe that one syphilitic poison does in different states of constitution, and under different circumstances, give rise to different secondary eruptions. We will not enlarge upon this point at present, because, in a future number of this Journal, we shall probably draw attention to the conditions which seem to us to modify the eruptions in question.

Mr. Judd is not quite correct in supposing that the same morbid poison always produces similar sequelæ. If a family, for example, are exposed to the cause, whatever that may be, of scarlet fever, the symptoms displayed by different members of the family are very various. One will have "putrid sore throat," and no eruption, and will die in a day or two—a second will have sore throat, dark-coloured eruption, and typhoid symptoms, and die also in a short time—a third will have very vascular sore throat, bright eruption, and highly inflammatory symptoms—a fourth will have a slight eruption, with scarcely any, or no perceptible sore throat—and several others may have slight sore throats, and no eruption at all. Who has not seen all these varieties of disease in a single family, when scarlet fever was prevailing? We feel no greater difficulty in supposing that two or three forms of cutaneous eruption may follow one syphilitic poison, than that diseases so opposed in their appearances, their phenomena, and their results, as a slight sore throat and a putrid scarlet fever, should arise from the application of one scarlatinal poison.

But Mr. Judd goes farther than we yet have followed him. The extent of his opinions may be gathered from the following quotation :—

"Yet that the primary sore is only the proximate or secondary cause of the eruption is clear, for does not the sore always, or next to always, follow inoculation by the poisonous virus, and precede the venereal eruption? That the sore and the effects following it are very intimately connected,—and more, that the eruption produced, and the sore producing it, bear a reciprocal influence over each other,—is demonstrated by such facts as the following :—A patient, now under my care, had, some weeks before, excoriated margins of the mouths of the sebaceous lacunæ around the corona glandis; and, after the usual lapse of time, they were followed by the puniceous patch eruption, which took place by the time the sore had healed without mercury. Shortly after this, he was attacked by pains in his limbs, and a considerable aggravation of all his secondary symptoms. The sympathizing cicatrix of the sore immediately re-ulcerated, and in so doing it assumed exactly the lozenge shape, and even the size of the puniceous patches, that were at the time declining from his skin. On the 30th of October, 1833, I examined the above-described sore; it had re-cicatrized, but it had left an elevated square surface, with a flat summit, standing up more than a line above the level of the surrounding integuments of the glans and foreskin, but still bearing its full resemblance to the puniceous patch eruption; for the remains of it being still visible upon his skin readily enabled me to compare them. In the case of James T——s, after the primitive sore had healed, lichenous puniceous patches broke out all over his person; and no sooner did the eruption commence, than the original cicatrix of the sore, sympathizing with the general inflammatory state of the cuticle, re-ulcerated, and formed one of those well-marked, elevated, lozenge-shaped, crimson surfaces, very similar indeed to the vivid patches of the eruption existing on his skin.

In a third case of puniceous patch eruption that I have at present under care, in T. J——th, the primitive sore left an immense elevated lump of induration, which felt like a piece of cartilage in the surrounding cellular membrane; yet the figure even of this induration was also a squarish oblong, much resembling in its shape and elevation the patches that formed his eruption.

(December.)—About two months afterwards, this person had another eruption of vesicular lichen; the indurated surface left by the sore was attacked by three small vesicles or ulcerations, and, on healing, they left three projections of the cuticle, not exactly like, to be sure, but little different from lichen in their height, colour, and shape." 155.

It is right to add to the preceding observations, the explanatory statement which succeeds it.

" However, it must not be expected that all primitive sores perceptibly exhibit on their surface the various changes that correspond with their eruptions, as such effects are only well marked at times, and not in all cases; probably from the original surface generally sloughing away, or being destroyed by caustic, and other applications, used to expedite the healing of the sore." 157.

The circumstance alluded to by Mr. Judd is undoubtedly the exception, and not the rule. Secondary eruptions occur upon the penis, as on other parts of the surface of the body. But the cicatrix of the primary sore very seldom indeed becomes affected in a manner corresponding to the secondary eruption which appears. A slight consideration must make it evident that this would be improbable. The secondary eruption is the evidence and the result of the contamination of the system. That being once contaminated, the primary sore has no more to do with the matter, and can only share the general infection. In the chapter of chances its site may be attacked with the secondary eruption as well as other parts, nay, as the cicatrix is frequently unhealthy, it might perhaps be rather more prone to take on a morbid action than contiguous sound skin.

Whether this be or be not the true explanation of the circumstance, we can affirm as a general fact, that the cicatrix does not take on an action, correspondent to the kind of the eruption. Now and then it re-ulcerates, but even this is not common. Usually it remains indurated, and the skin is rather red.

The second chapter of this second part, is " on the variety of absorbent surfaces and primary sores, and on the various modes by which the poison enters the system."

Mr. Judd points out, of course, the route of the absorbents, as the ordinary channel by which syphilis enters the constitution. On this we need not dwell. But it appears to us that Mr. Judd does not sufficiently distinguish between the inflammation of the absorbent, which must be the result of simple irritation, and that which is the result of the specific poison. If we see a slight abrasion produce inflammation of the absorbent vessels, and suppuration in the glands, we need not be surprised if a sore should occasion the same effects, independently of the action of a peculiar poison. No doubt this is frequently the case in syphilis, and we are confident that it is generally, if it is not always the case in gonorrhœa. We are occasionally consulted by a gentleman who scarcely ever has connexion without some enlargement of the inguinal glands ensuing. Purgatives, rest, and evaporating lotions always remove the irritation and the swelling.

It is a curious, but a well ascertained fact, that bubo occasionally precedes the appearance of a sore. If we trusted to the representations of patients, we should look upon this as a common occurrence, for they constantly overlook slight sores. But, discarding their testimony, we have occasionally, as we observed, opportunities of witnessing the fact ourselves. Probably in many of these cases the bubo is the product of simple irritation; that, at least, is the most reasonable view of the case. But in other instances, it is more rational to suppose that, in some way or other, the absorbent has received the poison independently of a breach of surface. The following memorandum on the subject by our author deserves consideration.

"In the month of June, 1818, I saw and made notes of three cases of buboes which took place from ten days to three weeks after connexion, without the parties having had any sore, by which it would be observed or suspected that the virus had entered their systems; but in one of them a deep sore, of a kind that would be termed chancre, appeared a fortnight after the bubo had formed, and at that very time too when the patient had a sore mouth from his gums and system being fully under the influence of mercury.

Another case of primary sore, forming more than two months after the bubo, will be seen in the case of James G——y, followed by maculæ cupreæ." 166.

Mr. Judd relates a case in illustration of these remarks; but we cannot look on it as very satisfactory.

*Case.* James G——y, June 14th, 1819.—He had connexion, and three weeks after it the glands in both groins began to enlarge; and getting irritated by his continuing to take his usual portion of exercise, they soon suppurated. In the latter end of August, a small sore made its appearance on the penis, over the mouth of a sebaceous lacuna; it looked as if he had rubbed the skin off. It healed several times, and again broke out, much induration occurring about it. His mouth was kept sore from the 17th of October until the 11th of November. During this time a slight crop of ecthyma made its appearance on the shoulders and back. In November, the patient leading a very irregular life, the groin re-inflamed and suppurated again. In September, 1820, *ten months afterwards*, he returned with several sinuses in the groins, and the lacuna, where the abrasion had been, was red and pouting. He had now some yellow marks on his skin.

"October 1st.—A number of very peculiar copper-stains, SPILI CUPREI, made their appearance in the skin of the lower part of his neck, breast, and shoulders. These stains did not come out all at once, but by a few at a time, in various forms; some as single spots, others coalesced in groups.

Fresh maculæ keep appearing every few days: those that came out first are desquamating, and throwing off branny scales, whilst fresh ones are appearing on other late healthy portions of skin, and there forming large patches of some inches in magnitude, especially upon the chest and axillæ." 433.

The copper-stains increased, and they felt as if the cuticle over them was slightly elevated. He was kept "under a long and steady course of mercury," without any influence on the eruption, and two years afterwards it was visible still.

This case is on many accounts unsatisfactory. In the first place, we have only the statement of the patient that the buboes occurred without any previous excoriation. In the second place, it is extremely dubious whether the eruption of ecthyma, which appeared upon the back and shoulders, was venereal. In the third place, the interval of ten months which elapsed between the patient's leaving the hospital and returning with the copper-stains, must render the source of all subsequent symptoms vague; for, during that time he was probably exposing himself continually to fresh infection, whilst an irritable, if not excoriated surface, was ready-formed to receive it. And, in the fourth place, it is more than probable that the copper-stains were not syphilitic at all, but an eruption of pityriasis. We are led to this opinion on two accounts—first, that the description and the plate of Mr. Judd, apply to pityriasis, and, secondly, that a long course of mercury had no influence on the eruption, which is constantly the case with that affection. On all these grounds, we cannot consider the case before us as free from many serious objections. We make these observations in order to shew that great caution must be exercised in forming opinions with respect to venereal infection and to secondary venereal eruptions. We never can expect to arrive at satisfactory conclusions on the characters, the course, or the treatment of syphilis, unless we are extremely sceptical in admitting evidence. We know of no disease in which more rigid reasoning is necessary, in order to dispel the clouds of uncertainty and error that hang over it.

Mr. Judd makes some remarks on venereal buboes, which are open to the objection we have already hinted—that he does not make the due allowance for ordinary irritation and inflammation of the absorbent or its gland. It does not follow, that, because an inguinal gland is enlarged, the venereal poison has been the cause of it. Herpes preputialis, occurring independently of all connexion, will at times occasion bubo. No doubt that bubo is often produced by the absorption of the virus; but, no doubt, the absorption of the virus has often nothing to do with it. It may be asked, how we discriminate bubo occasioned by simple irritation, from bubo occasioned by the ingress of a poison into the absorbent. In many instances the discrimination is impossible, and it is only a review of all the circumstances in the individual case, that can enable us to arrive at a correct or a probable opinion.

Mr. Judd observes that, when the sore or the glands are attacked by gangrene, and slough out, it is not more than once in five or six times that any venereal contamination takes place. The cases we have seen induce us to regard this, which is a commonly received opinion, with some doubt. From what we have observed, we should say that the occurrence of secondary symptoms is much influenced by the period at which the sloughing occurs. It is not unusual for patients in the lower classes to neglect venereal sores at their commencement. They go on for some time unattended to and unchecked, and then from intemperance, or cold, or mercury, or some debilitating agency, they slough. Under these circumstances, secondary symptoms are far from rare, and some of the very worst cases of secondary affections of the throat and skin which we have witnessed, have originated in this manner. But where sloughing occurs early, we have noticed, as, indeed, the majority of surgeons have done, that the frequency of secondary symptoms is diminished.

Mr. Judd alludes to the opinion of Mr. Hunter, that pus from a suppurating bubo will not occasion syphilitic disease. Mr. J. observes that he "may safely affirm that secondary symptoms would follow the entry of the pus from a bubo, either into the system of its producer, or into that of any other person." We agree with our author in the improbability of the absolute dogma of Mr. Hunter, but we could have wished that Mr. Judd had tested the matter experimentally. Half-a-dozen experiments would prove more than as many volumes of reasoning; it is a point which can only be decided by experiment.

We may proceed to the classification of sores proposed by Mr. Judd. It is contained in the following tabular view, and we must present it in the form and the words of our author, omitting some references to individual cases.

"*A Tabular View of the several Primary Sores and Appearances on the Penis that followed various Venereal Contaminations.*

1.—A SUPERFICIAL REDNESS* on the surface of the penis, either surrounding the contaminated mouths of the sebaceous lacunæ, or encircling the corona glandis, followed by absorption of various poisons, and by various eruptions.

2.—A HALF-EXCORIATED CRIMSON SURFACE on the glans, at times surrounded by semicircular radiated lines (and there may be traced occasionally an inflamed absorbent from it), followed by small vesicles on the brow and chin.

VAR.—Excoriations, fissures, and abrasions, most common between the glans and prepuce, forming absorbent surfaces, giving rise to abscesses in the body of the penis, and a great variety of secondary symptoms, as puniceous patch eruptions, sore throat, nodes, and inflamed synovial membranes.

3.—A PIMPLE, or pimples, more or less risen; or an oviform red surface on the glans, well corresponding with, and frequently followed by lichen.

VAR.—At times a pimple becomes vesicular, or suppurates, or breaks, and ulcerates. It generally produces bubo, pains, sore throat, lichen, ecthyma, iritis, opacities of the cornea, and thickened periosteum.

4.—A VESICLE on the glans or prepuce that appears about the third day after connexion, and leads to pains, pleuritis, puniceous and vesicular eruptions, rupia, ulcerated tonsils and pharynx, nocturnal pains, inflamed synovial membranes, corona veneris, tubercles, and psoriasis.

VAR.—Small vesicles that concrete or incrust, followed by pains in the shoulders; by sore throat; vesicles on the face and scalp; and also by psoriasis; and swelled legs and ancles.

VAR.—A circle of small vesicles on the glans and dorsum, producing pains, sore throat, clefts in the tongue, and general concentric vesicular eruptions.

VAR.—Herpes præputialis, or a cluster of small vesicles, set on risen inflamed bases, situated on the glans, prepuce, or body of the penis; or thigh, pubes, scrotum, or buttocks; followed by sore throat, pains in the knees and ancles, all respectively recurring for years in succession.

5.—A PUSTULE, or more, various in size, appearing from the third to the seventh day after connexion on the glans, or between it and the foreskin; on the body of the penis, pubes, or scrotum, and sometimes forming an indurated annulus, and followed by urethritis, pustular sore throat, pains, ecthyma, and suppurations in the submaxillary glands, and crural absorbents and iritis, or ulcerated cornea.

VAR.—Several smaller and less opaque pustules on the dorsum, about the fifth day after connexion, followed by pains, ecthyma, iritis, and ulcerated cornea, as it is with syphilitic urethritis and pustule; or abscess, or ulcers produced by it, within the urethra, come properly under this head, producing pustular eruptions and secondary symptoms.

6.—A CHANCRE or small deep round ulcer, with hardened edges. It generally appears about the third day after connexion; often healing by the tenth; is at times situated on the mouth of an absorbent; at times followed by nocturnal pains; at times, by effusion into the synovial membranes, with, sometimes, nodes and corona veneris; enlarged bones and testicles, boils, tubercles, lepra, and psoriasis.

7.—AN ULCER with a raised and glassy-looking level surface, spreading by a dark film at its edges, running perhaps half-way round the prepuce; and followed by bubo, puniceous patch eruption, or tubercles.

---

* "Every abrasion, pimple, herpes, pustule, wart, or slough, is capable of causing enlarged glands or buboes, in addition to the above symptoms.—In rare instances, absorption of virus takes place without breach of surface, or any visible mark."

8. A CLEAN SMALL ULCER, with a level base, situated on the glans a little deeper than the skin, followed by bubo and maculæ.

9.—A PHAGEDÆNIC BLEEDING ULCER,† beginning with a black spot on the penis, or cellular membrane, eating through the prepuce, or into the glans and corpora cavernosa, followed by puniceous patch eruption, pains, ulcerated tonsils, tubercles, and lepra.

VAR.—Healthy sores, changing their state; which at times increase by white sloughs, and often run into hospital gangrene."

The principal reasons of our author for supposing that he has arrived at an approximation to the correct enumeration and classification of venereal sores, will appear in the following passage.

" In limiting the number of sores and primary affections to about nine, (but without including their modifications,) I do imagine that I have come very near to the true number met with in England, Scotland, and Ireland: for, from the numerous drawings I had made of sores, and sketches of various venereal eruptions, and from the many manuscript accounts I have collected during a period of near twenty years, I can assert with truth, that on summing up all the various appearances of the sores, and comparing them and the number of the eruptions, I actually found their numbers exactly corresponded. At this I rejoiced, and hailed it as a happy criterion, showing that I had approached as near to the true number as the difficulty of the subject, the features of sores, and the variety of cutaneous affections probably admit of." 185.

We will now return to the arrangement of venereal sores, proposed by Mr. Judd. In the first place we may observe, that we presume the great object of any arrangement is the definition and description of venereal sores. As it is obvious that the penis is subject, like other portions of the surface of the body, to the ordinary affections of that surface, it is clear that they ought, if possible, to be separated from those specific venereal sores, which result from the application and peculiar influence of a morbid poison. Unless some such plan is adopted, we open the door to a dangerous latitude and laxity of doctrine, and we might as well declare, without circumlocution, that every consequence of sexual intercourse, from the simplest irritation or abrasion upwards, constitutes the venereal disease.

When we contemplate the "tabular view" of sores, which is spread before us, we are struck with the fact, that if it be correct, there is no affection of the surface of the penis, which must not, or may not be deemed syphilitic.

Our author's first variety of sore is a superficial redness on the surface of the penis, either surrounding the mouths of the sebaceous follicles, or encircling the corona glandis. If this be considered syphilitic, then many an honest man has syphilis, who never dreams of his condition, and many a case of such syphilis is cured, by a purge and a little goulard water. We do not say that such superficial redness may not, in some rare instances, indicate the operation of the syphilitic virus. But we are sure that in the great majority, it must be looked on as the effect of simple irritation, and that the surgeon who considers and treats it as syphilis, will greatly overrate its importance.

The second variety is a half-excoriated crimson surface on the glans.

† " Black and white phagedæna.—Both vesicles and pustules generally appear to the eye to commence as pimples, but soon either assume their proper form, or ulcerate.—At times, a peculiar half-excoriated red wart leads to bubo, contamination, and puniceous patch eruption."

Two cases are brought forward in support of the opinion, that this is a syphilitic affection.

*Case* 1. Thomas D——ge. August 30th, 1819.—" A small sore on the inside of the prepuce, which came a few days after connexion ; its margin is a little elevated ; of a deep red colour, and fissured." On the 8th of September were two other sores, like pimples, on the lower part of the glans. He was put on a course of mercury, and the sores were nearly healed, while two buboes were slowly supurating, when, on the 17th of October, it is stated that " the side of the urethra and glans are ulcerated in a semi-circular form, radiated in lines, and becoming very hard. There is another fresh small sore, with elevated edges on the glans inside the prepuce ; and around it, a red half-excoriated surface that looks as if it was formed of straight, crimson, radiant lines, leaving a healthy light surface between the inner edge of the red semi-circle and the sore." On the 20th of November, a vesicular eruption appeared on the face and nose. It dried up in ten days, and the patient then became well.

We do not perceive how this case establishes the correctness of the proposition embodied in the notice of the second variety of venereal sore. The " half-excoriated crimson radiated surface," was not the primary sore in this instance, and it is the primary sore from which we usually and properly date consecutive contamination. The radiated surface alluded to, was not only secondary in point of date, but appeared after the patient had been brought under the influence of mercury—a powerful disturbing circumstance. We are not convinced that the vesicular eruption on the face was syphilitic. Such eruptions are frequent, independently of syphilis, and its final disappearance in ten days is not the usual characteristic of secondary venereal ulceration.

*Case* 2. C. S——ll, April 25th, 1823.—" A sore formed on what appeared to be the orifice of one of the lacunæ of a sebaceous gland, (or the mouth of an absorbent,) in the depression between the glans and body on the right side of the penis. A defined red line was clearly traced from the sore, running under the skin, across the body of the penis, from its right side towards the left groin, and in a few more days a gland on that side formed bubo, which suppurated, and was followed by vesicles on the forehead."

How this case proves, or even bears upon the question, we confess ourselves unable to perceive. No mention is made of the nature of the sore—nor is any evidence of the venereal character of the vesicles on the forehead offered.

*Case* 3. C. K——by. January, 1819.—" He has a number of sores that look like tears, situated between the glans and body of the penis, and around the foreskin : he states that they commenced a few days after connexion." He had several abscesses on the pubes and the penis. They formed sloughy sores. Mercury was given, but it had not much effect.

We are equally unable to perceive the applicability of this, as of the former case. Sores " looking like tears" can hardly be arranged under the head of " a crimson excoriated surface."

We have not sufficient space to consider seriatim, each variety of venereal sore that follows. Our observations must therefore assume a more general character and application.

The objections to the classification of our author may be shortly stated. In the first place it is too vague ; and, in the second, it is imperfect. It is too vague, because appearances are laid down as venereal, and as leading to secondary contamination, which the common experience of the whole profession has decided to arise frequently without the agency of the venereal virus. The fourth kind of sore, for instance, is said to be a vesicle, more or less risen, that leads to pains, pleuritis, and so forth; while its sub-varieties consist of "small vesicles that concrete or crust"—a "circle of small vesicles on the glans and dorsum penis"—and "herpes præputialis." Now every surgeon of even moderate experience must have witnessed these appearances, when no sexual intercourse had taken place. They cannot, therefore, be looked upon as specific consequences of the syphilitic virus. If superficial redness, an excoriated surface, a pimple, a vesicle, and a pustule, may each and all occur, as undoubtedly they may and do, independently of syphilis, how can the surgeon pronounce on their syphilitic character? Yet these are the first five primary sores of Mr. Judd. The error into which he has fallen has been to take primary venereal affections at a period when they have not yet assumed distinctive characters. If the syphilitic virus produces a vesicle, a pimple, or a pustule, we are not to assume that all vesicles, pimples, and pustules are syphilitic, because these are general characters, common to the tribe of eruptive disorders, whether venereal or not. The vesicle or pustule may or may not be syphilitic, and it is impossible to determine from any investigation of its features, whether it is so or not. We must wait until the poison has produced more specific and distinct appearances—until, in short, a surface or an ulcer has occurred, which possesses characters peculiar to itself. This is the method which has been pursued by all inquirers, and although there are some disadvantages attendant on it, it is the only one that offers a chance of our arriving at definite results.

The classification before us is imperfect, for while it has admitted pimples and vesicles of dubious character, it has excluded two or three decided and not unfrequent forms of venereal ulceration. The multifarious fungating sore—the condylomatous sore—and some of those peculiar creeping sores, which cannot be fairly included in the broad term of phagedæna, find, if we read this classification rightly, no home within its precincts.

We have remarked that Mr. Judd makes little reference to syphilis as it appears in women. As an army-surgeon, his opportunities have been chiefly in the military hospital or camp, yet we feel assured that, unless the disease is extensively watched in both sexes, it cannot be well understood or described. It is only in a large Lock Hospital that venereal maladies can be adequately studied.

In our next number we shall pass to the secondary effects of lues, and complete our notice of the present work.

